# A multi-society Delphi consensus statement on the diagnosis of familial chylomicronemia syndrome

**DOI:** 10.20945/2359-4292-2025-0071

**Published:** 2025-09-24

**Authors:** Carlos A. Aguilar-Salinas, Rodrigo Alonso, Gabriela Berg, Alejandro Alberto Castellanos Pinedo, Pablo Corral, Ivette Cruz Bautista, María Cristina Izar, Carlos O. Mendivil, Juan Patricio Nogueira, Alejandro Román-González, Raúl D. Santos, Hernando Vargas-Uricoechea

**Affiliations:** 1 Unidad de Investigación de Enfermedades Metabólicas Instituto, Nacional de Ciencias Médicas y Nutrición Salvador Zubirán, México City, México; 2 Dirección de Investigación, Instituto Nacional de Ciencias Médicas y Nutrición Salvador Zubirán, México City, México; 3 Tecnológico de Monterrey, Escuela de Medicina y Ciencias de la Salud, México City, México; 4 Centro Avanzado Medicina Metabólica y Nutrición, Santiago de Chile, Chile; 5 Laboratorio de Lípidos y Aterosclerosis, Facultad de Farmacia y Bioquímica, Universidad de Buenos Aires, Buenos Aires, Argentina; 6 Universidad del Sinú Montería, Córdoba, Colombia; 7 Hospital San Jerónimo de Montería, Córdoba, Colombia; 8 Universidad FASTA, Mar del Plata, Argentina; 9 Instituto de Investigaciones Clínicas, Mar del Plata, Argentina; 10 Unidad de Investigación de enfermedades metabólicas, Departamento de Endocrinología y Metabolismo, Instituto Nacional de Ciencias Médicas y Nutrición Salvador Zubirán, Mexico City, Mexico; 11 Universidade Federal de São Paulo, São Paulo, SP, Brasil; 12 School of Medicine, Universidad de los Andes, Bogotá, Colombia; 13 Endocrinology Section, Department of Internal Medicine, Fundación Santa Fe de Bogotá, Bogotá, Colombia; 14 Universidad Nacional de Formosa, Formosa, Argentina; 15 Universidad de Antioquia, Medellín, Colombia; 16 Instituto do Coração da Clínica Lipídica, Hospital das Clínicas da Faculdade de Medicina da Universidade de São Paulo, São Paulo, SP, Brasil; 17 Hospital Israelita Albert Einstein, São Paulo, SP, Brasil; 18 Grupo de Investigación en Enfermedades Metabólicas, Departamento de Medicina Interna, Universidad del Cauca, Popayán, Colombia

**Keywords:** familial chylomicronemia syndrome, FCS, hypertrigliceridemia, Latin America, lipoprotein lipase

## Abstract

**Introduction:**

Familial chylomicronemia syndrome (FCS) is an autosomal recessive disorder that affects approximately 1 to 10 individuals per million and is caused by variants in the genes encoding for the lipoprotein lipase (LPL) enzyme. In addition to its heterogeneous clinical presentation, FCS is characterized by a higher risk of life-threatening, recurrent acute pancreatitis and type 3 diabetes. Since available evidence on FCS in Latin America is limited, there is a clear need for a consensus document that provides relevant recommendations to guide the management of suspected cases and optimize disease diagnosis across the region.

**Methods:**

A panel of specialists from Latin America with extensive experience in the diagnosis of chylomicronemia was invited to participate in the creation of this document. The modified Delphi technique was used to reach group consensus through multiple rounds of questionnaires using statistical techniques and controlled feedback. Results and discussion: Seventeen recommendations on diagnosis of FCS were generated. This consensus reflects the collaborative efforts of Latin American scientific societies and is essential to suspect and diagnose FCS. The organizations that support this document, including Sociedad Argentina de Lípidos, Federación Argentina de Sociedades de Endocrinología, Fundación Bioquímica Argentina, Corporación Grupo Chileno de Trabajo en Ateroesclerosis, Asociación Colombiana de Endocrinología, Diabetes y Metabolismo, Departamento de Aterosclerose da Sociedade Brasileira de Cardiología, and Sociedad Mexicana de Nutrición y Endocrinología, are a robust support network that might aid the adoption of these recommendations in local healthcare systems.

## INTRODUCTION

Fasting plasma triglyceride (TG) levels are normally below 150 mg/dL and may increase as a result of multiple factors, including but not limited to diet, obesity, and diabetes, which are known to interact with polygenic determinants. However, extreme levels of TGs are rarely explained by monogenic defects, such as familial chylomicronemia syndrome (FCS) (^1^). FCS is an autosomal recessive disorder that affects approximately 1 to 10 individuals per million and is caused by biallelic pathogenic (P) or likely pathogenic (LP) variants in the genes encoding the lipoprotein lipase (LPL) enzyme. In addition to its heterogeneous clinical presentation, FCS is characterized by a greater risk of life-threatening, recurrent acute pancreatitis and type 3 diabetes (^2^). Since available evidence on FCS in Latin America is limited (^3^-^6^), there is a clear need for a consensus document that provides relevant recommendations to guide the management of suspected cases and optimize disease diagnosis across the region.

## METHODS

Evidence-based medicine approaches have significantly contributed to the development and improvement of clinical practice guidelines. Nevertheless, data interpretation and extrapolation to other sociocultural contexts may hinder their implementation in everyday practice. The modified Delphi method has proven to be a valid and reliable strategy that can help overcome these barriers by facilitating the collection of expert opinions to gain consensus on a specific topic (^7^).

A panel of specialists from Latin America with extensive experience in the diagnosis of chylomicronemia were invited to participate in the creation of this document. The modified Delphi technique was used to reach group consensus through multiple rounds of questionnaires using statistical techniques and controlled feedback (^8^,^9^).

A questionnaire consisting of 30 items (18 with Likert response options, 7 with polytomous response options, and 5 with free-text response options) was drafted on the basis of available evidence from the main biomedical databases and shared with the expert panel via a digital platform to maintain the blinding and anonymity of the responses. The levels of agreement were defined as follows:

**Strong (very high) expert consensus:** ≥80% agreement, irrespective of the abstention rate;

**Expert consensus:** ≥70% agreement + <20% abstention rate.

Some questions were rephrased on the basis of suggestions from the expert panel when no consensus was reached in the first round. The revised questions were submitted for reconsideration in successive rounds. Stability of the consensus was achieved if ≥70% of the responses remained unchanged from previous rounds. To conclude the process, a virtual meeting was held to agree on a final version containing 17 recommendations and statements, including a point of good practice, primarily based on experience.

## RESULTS

### Recommendation/statement #1

According to the panel of experts, *FCS* is the preferred acronym for “familial chylomicronemia syndrome”. Expert consensus recommendation/statement (agreement: 75%).

Given the broad spectrum of potential symptoms in affected individuals, this condition is better characterized as a “syndrome” (^10^). While some patients present with mild or no symptoms, the diagnostic process begins with the detection of severe hypertriglyceridemia (HTG). Although several cases are seemingly sporadic as a result of the recessive inheritance pattern of the disease, several organizations, such as the Lipoprotein Lipase Deficiency (LPLD) Alliance and the FCS Foundation, have pragmatically used the term “familial” when referring to FCS (^10^).

### Recommendation/statement #2

On the basis of available evidence and experience, the expert panel recommends the use of clinical scores for the diagnosis of FCS. Strong expert consensus recommendation/statement (agreement: 100%).

FCS is an autosomal recessive disorder of chylomicron metabolism marked by a substantial increase in circulating TG levels. A poor response to conventional lipid-lowering therapies is common among these patients because of impaired lipid clearance. However, FCS remains largely underdiagnosed, which may lead to delayed initiation of adequate treatment (^11^). Available pragmatic scores for the diagnosis of FCS, which rely mainly on clinical parameters (**Tables 1** and **2**), may assist in differentiating between FCS and multifactorial chylomicronemia syndrome (MCS) (^10^).

**Table 1:** Mouline score (^10^)

Criteria	Score
Fasting TG levels >885 mg/dL on at least three consecutive measurements (^*^) Fasting TG levels >1,770 mg/dL at least once	+5 +1
Previous TG levels <177 mg/dL at least once	-5
Absence of secondary factors (†) (except for pregnancy (‡) and use of ethinylestradiol)	+2
History of pancreatitis	+1
Unexplained recurrent abdominal pain	+1
No history of familial combined hyperlipidemia	+1
No response (TG levels decreased by <20%) to lipid-lowering treatment	+1
Age at symptom onset <40 years <20 years <10 years	+1 +2 +3
Score ≥10: FCS is very likely Score ≤9: FCS is unlikely	Score ≤8: FCS is very unlikely

FCS: familial chylomicronemia syndrome; TG: triglyceride.

(^*^) Measurements performed at least one month apart.

(†) Alcohol, diabetes, metabolic syndrome, hypothyroidism, corticotherapy, and the use of other drugs.

(‡) If the diagnosis is made during pregnancy, a second test will be required to confirm the diagnosis postpartum.

**Table 2:** Clinical scores suggested by Hegele and cols. (^12^)

Criteria	Score
Age at first visit Less than 1 year 1 to 9 years ≥10 years	0 +12 +12 (^*^)
BMI <25 kg/m^2^ or <85^th^ percentile ≥25 kg/m^2^ or ≥85^th^ percentile	+9 0
History of pancreatitis Present Abdominal pain without pancreatitis No abdominal pain or pancreatitis	+16 +9 0
Secondary factors that may trigger HTG None At least 1	+11 0
Lab tests TG levels are always >880 mg/dL TG/TC ratio >8 ApoB100 <1 g/dL	+13 +8 +12
Additional scores Aged 1 to 9 years + no secondary factors TG/TC ratio >8 + ApoB100 <1 g/dL TG/TC ratio >8 + no secondary factors	+7 +7 +5
**If score is ≥60, consider clinical diagnosis of FCS**	

ApoB100: apolipoprotein B-100; BMI: body mass index; HTG: hypertriglyceridemia; TC: total cholesterol; TG: triglyceride.

(^*^) If the age of onset of HTG was younger than 10 years.

### Recommendation/statement #3

On the basis of available evidence and experience, the expert panel suggests that the following parameters should be considered when establishing the clinical diagnosis of FCS:

[3.1] Absence of secondary factors for severe HTG. Strong expert consensus recommendation/statement (agreement: 100%).

[3.2] History of pancreatitis. Strong expert consensus recommendation/statement (agreement: 100%).

[3.3] Refractoriness to conventional treatments for HTG. Strong expert consensus recommendation/statement (agreement: 100%).

[3.4] A history of unexplained recurrent abdominal pain due to other reasons.

Strong expert consensus recommendation/statement (agreement: 100%).

[3.5] Early disease onset (childhood, adolescence, or young adulthood). Strong expert consensus recommendation/statement (agreement: 100%).

[3.6] Consistent lipid profile (normal or low levels of direct low-density lipoprotein cholesterol [LDL-C], low levels of high-density lipoprotein cholesterol [HDL-C], TG/total cholesterol (TC) ratio >5, and low-to-normal levels of apolipoprotein B [apoB]). Strong expert consensus recommendation/statement (agreement: 100%).

[3.7] Onset of diabetes following the diagnosis of HTG. Expert consensus recommendation/statement (agreement: 75%).

The selection of these parameters, supported by evidence and the experience of the group of experts, is in line with a recent Delphi-based consensus involving specialists in the management of FCS patients. Factors such as age at onset, history of abdominal pain or pancreatitis, TG/TC ratio, and apoB levels were also considered relevant for the clinical diagnosis of FCS (^12^). In light of the ongoing epidemic of overweight and obesity, a normal body mass index (BMI) might serve as a useful indicator; however, both normal-weight and overweight individuals can develop FCS (^13^).

### Recommendation/statement #4

On the basis of available evidence and experience, the expert panel suggests that the following genes should be prioritized when screening patients for mutations to confirm the genetic diagnosis of FCS:

[4.1] *LPL.* Strong expert consensus recommendation/statement (agreement: 91.7%).

[4.2] *GPIHBP1.* Strong expert consensus recommendation/statement (agreement: 91.7%).

[4.3] *APOA5.* Strong expert consensus recommendation/statement (agreement: 83.3%).

[4.4] *APOC2.* Expert consensus recommendation/statement (agreement: 75%).

[4.5] *LMF1.* Expert consensus recommendation/statement (agreement: 75%).

Depending on the target population, 50-90% of patients with FCS may have biallelic variants in the *LPL* gene (^14^) encoding the LPL enzyme that mediates the lipolysis of TGs in chylomicrons, very low-density lipoproteins (VLDLs), intermediate-density lipoproteins (IDLs), and other particles. The remaining 10-50% of cases have biallelic mutations in one of the other four “canonical” genes encoding cofactors and proteins that interact with LPL, including apolipoprotein C2 (*APOC2*), glycosylphosphatidylinositol-anchored HDL-binding protein 1 (*GPIHBP1*), apolipoprotein A5 (*APOA5*), and lipase maturation factor 1 (*LMF1*) (^11^,^15^-^17^).

### Recommendation/statement #5

On the basis of available evidence and experience, the expert panel states that, under ideal circumstances, genetic testing is the recommended method to confirm the diagnosis of FCS. Strong expert consensus recommendation/statement (agreement: 83.3%).

### Recommendation/statement #6

On the basis of available evidence and experience, the expert panel states that, under real-world circumstances, the clinical evaluation of the patient (including clinical scores) is the best diagnostic method for FCS. Expert consensus recommendation/statement (agreement: 75%).

In situations where access to genetic testing is restricted, clinical assessment plays a central role in the diagnosis of FCS.

### Recommendation/statement #7

On the basis of available evidence and experience, the expert panel notes that nearly 25% of patients presenting with clinical features consistent with FCS have a negative genetic test result (agreement: 75%).

### Recommendation/statement #8

On the basis of available evidence and experience, the expert panel considers that measuring LPL activity - if available - is the most adequate approach for patients with a clinical presentation suggestive of FCS who tested negative for monogenic mutations. Expert consensus recommendation/statement (agreement: 75%).

Point of good practice: this preferential recommendation does not preclude the use of other strategies, such as the identification of anti-*GPIHBP1* antibodies or whole-exome sequencing.

When the test can be performed, LPL activity is typically severely reduced among patients with FCS carrying a homozygous variant in the *LPL* gene, similar to patients with homozygous or heterozygous loss-of-function mutations in other canonical genes (^10^). Both the laboratory’s capabilities and availability should be considered when using this technique (^11^).

### Recommendation/statement #9

On the basis of available evidence and experience, the expert panel suggests that the LPL activity assay (if available) should be conducted in patients with clinical features and laboratory findings consistent with FCS + a negative genetic test or a variant of unknown significance (VUS). Strong expert consensus recommendation/statement (agreement: 100%).

If available, LPL activity is a reliable marker for the diagnosis of FCS in patients with HTG (^18^). In addition, a statistically significant correlation has been reported between the activity of this enzyme and diagnostic scores that include clinical and biochemical parameters (^19^).

### Recommendation/statement #10

On the basis of available evidence (sensitivity, specificity) and experience, the expert panel makes no recommendation for any particular method or threshold of choice when measuring LPL activity for the diagnosis of FCS (agreement: 100%).

Each laboratory should set their own cutoff values to measure LPL activity, which is typically ≤25% of said value among patients with FCS (^18^).

### Recommendation/statement #11

On the basis of available evidence and experience, the expert panel makes no recommendations concerning the utility of lipoprotein electrophoresis in patients suspected to have FCS (agreement: 100%).

### Recommendation/statement #12

On the basis of available evidence and experience, the expert panel recommends considering the factors listed below to reconsider the diagnosis of autoimmune chylomicronemia:

[12.1] Intermittent HTG. Strong expert consensus recommendation/statement (agreement: 91.7%).

[12.2] Inconclusive genetic testing results. Strong expert consensus recommendation/statement (agreement: 91.7%).

[12.3] History of other autoimmune conditions. Strong expert consensus recommendation/statement (agreement: 83.3%).

[12.4] Adult onset. Expert consensus recommendation/statement (agreement: 75%).

Autoimmune chylomicronemia can be caused by the presence of anti-*GPIHBP1* antibodies. This form of acquired chylomicronemia is usually characterized by intermittent HTG and may be associated with a previous autoimmune condition (including detectable antinuclear antibodies) (^20^).

### Recommendation/statement #13

On the basis of the available evidence and experience, the expert panel suggests that the diagnostic approach for FCS should not be changed under the following special circumstances: (a) patients hospitalized for acute pancreatitis; (b) pregnant patients; and (c) pediatric patients. Strong expert consensus recommendation/statement (agreement: 100%).

The diagnostic approach for patients aged under 10 years is comparable to that for adults (^21^). In addition, variables used for the clinical diagnosis of FCS also seem to be applicable to pregnant patients (^22^).

### Recommendation/statement #14

On the basis of available evidence and experience, the expert panel recommends considering a conclusive diagnosis under the following circumstances:

[14.1] Clinical, biochemical, and genetic testing consistent with FCS and absent LPL activity: diagnosis of FCS. Strong expert consensus recommendation/statement (agreement: 100%).

[14.2] Clinical and biochemical traits consistent with FCS + negative genetic testing + normal LPL activity: diagnosis of MCS. Strong expert consensus recommendation/statement (agreement: 91.7%).

In the context of this recommendation, the presence of heterozygous P/LP variants in a single canonical gene or the detection of homozygous or heterozygous VUS in the same genes are also regarded as negative genetic test results.

### Recommendation/statement #15

On the basis of available evidence and experience, the expert panel suggests that the possible diagnosis of FCS should be considered in patients with clinical and biochemical features consistent with FCS + negative genetic tests + low LPL activity. Strong expert consensus recommendation/statement (agreement: 100%).

### Recommendation/statement #16

On the basis of available evidence and experience, the expert panel suggests that the probable diagnosis of FCS should be considered in patients with clinical and biochemical traits consistent with FCS + inconclusive genetic tests + low LPL activity. Strong expert consensus recommendation/statement (agreement: 100%).

Differentiating FCS from MCS can be challenging. While ideally, the detection of pathogenic genetic variants is the preferred diagnostic method (recommendation/statement #5), genetic tests may sometimes yield negative results for canonical genes in nonresponders to conventional lipid-lowering treatments who present with phenotypic characteristics of FCS. Therefore, measuring LPL activity in these patients may help confirm the diagnosis (^19^). Notably, as new evidence continues to emerge, a series of genetic variants currently regarded as VUS might eventually be reclassified as pathogenic and, therefore, become diagnostic for the disease in the future.

### Recommendation/statement #17

On the basis of available evidence and experience, the expert panel suggests that the diagnosis of MCS should be considered in patients with clinical and biochemical traits consistent with FCS + inconclusive genetic tests + normal LPL activity.

Strong expert consensus recommendation/statement (agreement: 100%).

## DISCUSSION

FCS is a rare and intricate metabolic disorder that carries serious risks for patients, including recurrent pancreatitis and metabolic complications, such as type 3C diabetes. In Latin America, this condition entails major difficulties in diagnosis and treatment, mostly due to barriers in access to specialized diagnostic tests coupled with the heterogeneity of health care systems across the region.

The diagnosis of FCS is largely based on the identification of extreme and persistent elevations in plasma or serum levels of TGs (>885 mg/dL) as a consequence of genetic defects in LPL or its cofactors, which impair the metabolism of TG-rich lipoproteins. However, in Latin American countries, the availability of specific genetic tests is limited or even inaccessible due to resource and infrastructure limitations. In addition, the genetic diversity of this population contributes to the complexity linked to the genetic diagnosis of FCS; recent studies have reported that non-European ancestry might increase the likelihood of developing FCS (^23^), which may be subject to specific validations in the region. As a result, the implementation of diagnostic scores, as recommended by this consensus, might facilitate the standardization of criteria.

A further challenge concerning the timely diagnosis of FCS in Latin America is the lack of information on the prevalence of this syndrome in the region. Some local studies suggest a prevalence of 2.4% among patients with severe HTG in a Colombian region (^4^) or 3% in some areas of Argentina (^3^). This hampers the early detection of at-risk patients and emphasizes the need for real-world studies in the region to characterize this disease more accurately and adjust management strategies to the local context. The diversity and fragmentation of health care systems in Latin America is a major setback for the implementation of these consensus recommendations, along with the profound differences in terms of coverage and access to specialized diagnostic tests and treatments.

Moreover, the availability of diagnostic tests for FCS varies greatly across countries within the region. While genetic testing may be feasible in some referral centers in Argentina, Brazil, Chile, Colombia, and Mexico, these options are virtually nonexistent in other countries, and the determination of LPL activity is currently only available in Argentina. Restricted access to diagnostic tests interferes with the ability of health care professionals to confirm FCS (^24^), which may result in misdiagnoses and inadequate treatments that fail to address patients’ needs.

This consensus reflects the collaborative efforts of Latin American scientific societies and is essential for suspecting and diagnosing FCS. The organizations that support this document, such as *Sociedad Argentina de Lípidos, Federación Argentina de Sociedades de Endocrinología, Fundación Bioquímica Argentina, Corporación Grupo Chileno de Trabajo en Ateroesclerosis, Asociación Colombiana de Endocrinología, Diabetes y Metabolismo, Departamento de Aterosclerose da Sociedade Brasileira de Cardiología,* and *Sociedad Mexicana de Nutrición y Endocrinología*, form a robust support network that might aid the adoption of these recommendations in local health care systems.

Finally, cooperation within multicenter research networks and patient follow-up might offer critical insights into the clinical evolution of FCS in Latin American populations, an area where current knowledge is limited.

## CONCLUSIONS

FCS poses a significant challenge for health care systems in Latin America, both in terms of diagnosis and treatment.

The recommendations and statements outlined in this document have been created on the basis of the best available evidence, the opinions of the participating experts, and the endorsement of major scientific societies in Latin America and are intended as guidance for health care teams involved in the diagnosis of patients with suspected FCS. This consensus represents a step forward in the creation of a regional strategy that optimizes the diagnosis of FCS, establishes specific treatment protocols, reduces the risk of serious complications, and improves the quality of life of patients with FCS. The ongoing cooperation between regional scientific societies, the commitment to real-world research, and the introduction of accessible and cost-effective diagnostic solutions will be key to the success of this initiative in the coming years.

As with any document of this nature, periodic updates are recommended upon the emergence and dissemination of novel scientific findings and the growing experience of health care professionals.

**Disclosure:** nCarlos Aguilar Salinas receives grants from the Universidad of Monterrey. Rodrigo Alonso has received honoraria as speaker and member of advisory boards from Abbott, Boehringer-Ingelheim, Axon-Pharma Novartis, NovoNordisk, PTC Therapeutics, Recalcine, Saval and Teva. Gabriela Berg is an employee of the Universidad de Buenos Aires (UBA), has received grants from UBA and the *Agencia Nacional de Promoción Científica y Tecnológica* (Argentina) and honoraria as a speaker and grants for participation in a Congress from PTC Therapeutics. Pablo Corral has received honoraria as a speaker from Amgen, MSD, Novartis and PTC Therapeutics, and as an advisor from MSD, Novartis, NovoNordisk and PTC Therapeutics. Ivette Cruz Bautista has received honoraria as a speaker and/or member of advisory boards from Amgen, AstraZeneca, Boehringer-Ingelheim, Novartis, NovoNordisk and Silanes. María Cristina Izar has received honoraria as a speaker from BMS, Daiichi-Sankyo, GSK, Novartis, NovoNordisk, Sanofi and PTCBio; she has received grants from Amryt Pharma, PTC Bio, the *Conselho Nacional de Desenvolvimento Científico e Tecnológico* (Brazil) and the *Fundação de Amparo à Pesquisa do Estado de São Paulo*; she has been an advisor for BMS, Daiichi-Sankyo, Novartis, NovoNordisk and PTC Bio; she has been enrolled patients for clinical studies for Novartis and NovoNordisk. Carlos Mendivil is an employee of Universidad de los Andes; he has received honoraria as a speaker from Abbott, Amgen, AstraZeneca, Bayer, Beckman Coulter, Boehringer Ingelheim, Colmédica, Jannsen, MSD, Novamed, Pfizer, Procter & Gamble, and Sanofi; he has received honoraria as a member of advisory boards from Abbott, PTC Therapeutics, Procaps, Mitotherapies, NovoNordisk, Procter & Gamble and Team Foods; he has received honoraria as an investigator from Merck, Mitotherapies, PTC Therapeutics and Team Foods. Alejandro Román González is an employee of Universidad de Antioquia, has received grants from the *Asociación Colombiana de Endocrinología, Diabates and Metabolismo* has received honoraria as a speaker from Abbott, ACE, Amgen, Bayer, Biosett, Biotoscana, Ipsen, Pfizer, Recordati, Sanofi, Ultragenyx and Valentech. Raúl Santos has received grants from Amgen, Eli-Lilly, Esperion, Kowa, Ionis, MSD, Novartis, Sanofi/Regeneron and PTC Therapeutics; he has received an scholarship from the *Conselho Nacional de Desenvolvimento Científico e Tecnológico* (Brazil) and has received honoraria as a speaker from Amgen, Boehringer-Ingelheim, Daiichi-Sankyo, Eli-Lilly, Novartis, Novo-Nordisk, Libbs, Sanofi/Regeneron, Torrent and Ultragenyx. Hernando Vargas-Uricochea has received honoraria for enrolling patients for a clinical study by Sanofi and has received honoraria as a speaker from Abbott, Sanofi and PTC Therapeutics. Juan Patricio Nogueira and Alejandro Castellanos Pinedo have no conflict of interest to disclose.

## Data Availability

datasets related to this article will be available upon request to the corresponding author.
